# Modification of glass screen printed electrodes with graphene quantum dots for enhanced power output in miniaturized microbial fuel cells

**DOI:** 10.1038/s41598-024-80925-x

**Published:** 2024-12-02

**Authors:** Yuvraj Maphrio Mao, Khairunnisa Amreen, Rajnish Kaur Calay, Aritro Banerjee, Sanket Goel

**Affiliations:** 1 MEMS, Microfluidics and Nanoelectronics (MMNE) Lab, Birla Institute of Technology and Science (BITS) Pilani, Hyderabad Campus, Hyderabad, 500078 India; 2 Department of Electrical and Electronics Engineering, Birla Institute of Technology and Science (BITS) Pilani, Hyderabad Campus, Hyderabad, 500078 India; 3 Department of Mechanical Engineering, Birla Institute of Technology and Science (BITS), Pilani, Hyderabad Campus, Hyderabad, 500078 India; 4 Department of Building, Energy and Material Technology, The Arctic University of Norway, UiT, Narvik, 8515 Norway

**Keywords:** Graphene Quantum Dots, Glass Screen Printed, Miniaturized Microbial Fuel Cell, And Power Generation, Energy science and technology, Engineering, Materials science

## Abstract

This paper demonstrates screen-printing technique, Glass Screen printed (GSP) on glass layer with Graphene Quantum Dots (GQDs) via drop casting approach to manufacture electrodes for Miniaturized Microbial Fuel Cells (MMFCs). MMFCs are viable options to sustainably operate low-power devices such as sensors, implantable medical devices, etc. However, the technology is still not fully mature for practical applications due to limitations of output power. Materials and design improvements are required for decreasing internal resistance for better electron transfer and improving overall performance. In this work the electrodes manufactured by GSP technique, and anode modified by GQD was tested in MMFC using RO wastewater. It was found that the GQDs increased the surface area to improve electron transfer kinetics at the anode. As a result, GQDs-based GSPEs showed 7.4 times higher power output 332 nW/cm^2^ compared to its unaltered electrode which displayed a power output of 44.8 nW/cm^2^. Electrodes made by GSP technique are more durable and less susceptible to biofouling and corrosion compared to conventional methods. The modified anodes further showed sustained output for long term operation.

## Introduction

Microbial Fuel Cells (MFC) are bio-electrochemical fuel cell-based system that generates electricity from the microbial oxidation of organic material. Electricity is produced by naturally existing microbes called electrogene producing electrons during oxidation at anode (electron donor) which are driven through a circuit towards cathode (electron acceptor). Typical redox half reactions for a variety of disaccharides can be shown as

C_12_H_22_O_11_ + 13H_2_O⟶12CO_2_ + 48 H^+^+48e^−^ (at anode) and

12O_2_ + 48e^-^ + 48 H^+^ →24H_2_O (at cathode).

The main challenge in (MFC) technology is low power output and the high cost required to scale up the system to reach usable energy levels for large scale practical applications. It has been observed that smaller MFC reactors displayed lower activation losses, higher substrate utilization (mass transfer) and better diffusion of protons H^+^ out of the biofilm^1^ thus resulting in higher power output^2^. Thus, application of small or miniaturized MFC or MMFC is now becoming attractive for powering small portable power electronics, implantable medical devices, remote sensors without a need for recharging. However, cost effectiveness and performance are critical criteria for users of the technology. The electrodes in a cell conduct electrons and thus influence the overall performance of the cell.

For a MMFC electrode material must be biocompatible, conductive, and corrosion resistive, has good mechanical strength should be low cost for commercial competitiveness^11^.

Due to its favorable properties, carbon-based materials are the most commonly used for manufacturing electrodes. Metal-based electrodes are another option for electrodes, but they are highly prone to corrosion and not compatible with microorganisms^12^. To enhance the compatibility with microorganism, researchers have used composite-based metal or metal oxides for electrodes^13,14^. Many researchers are leveraging 3D printing technology for manufacturing electrodes which allows them to iterate various design and materials very fast to reach an optimal solution^15,16^. With 3D printing different shapes and porous structures to obtain large surface to volume ratio can be explored. Even though such approaches have been widely studied, there have been instances where the limitations have been displayed. As for the instances,

after a certain number of cycles, the 3D-printed electrodes show negative characteristics including biofouling due to the accumulation of microbial biomass on the surface of the electrodes which may hinder the repeatability and the overall performance of the operating MFCs^17,18^. While the metal/metal oxide-based electrodes displayed corrosive qualities due to the formation of acid on the surface of the electrodes because of the microbial byproducts^19^.

Choosing Glass Screen Printed Electrodes (GSPEs) as the layer/electrode material is another approach to tackle the limitation/restriction faced while operating with 3D printed polymer/metal/metal-oxide based electrodes. GSPEs can be easily sterilized using methods like autoclaving and dry heat eliminating the risk of biofouling improving and achieving a stable repeatability. GSPEs also possess a high degree of chemical stability which reduces the risk of electrode corrosion^20^. Table 1 summarizes the outcome from various reported MMFCs which are significantly smaller and miniaturized compared to the conventional MFCs. This miniaturization inherently reduces the scale of power output due to small surface area and reduced microbial activity. Additionally, the design is based on screen-printed conductive carbon ink layered on glass, which limits the power generation as compared to the larger system with complex architecture and materials. Despite all the limitations, the MMFCs contribute towards small-scale power applications and cost-effective fabrication techniques, particularly suited for scenarios where compactness and affordability are key considerations.

**Table 1:** Comparative study of various nanomaterial based MMFCs.

Electrode	Power density	Current density	Outcomes	Ref
Graphene screen printed electrodes	5.16 µW/cm^2^	16 µA/cm^2^	Direct Electron Transfer has been achieved by coating the gold nanoparticles and Graphene-based screen-printed electrons. The approach of modifying has also improved the overall performance of the enzymatic fuel cells operated during this work.	^3^
Carbon Veil Fiber	0.43 mW	–	The work demonstrated the power output of ceramic-based MFCs stacked and experimented with different dimensions and parameters and provided needful insights on the importance of stacking and its contribution to small-scale applications.	^4^
Carbon nanotubes	0.83 W/m^2^	2.59 A/m^2^	A small scaled mintuarized MFC with high volumetric density is fabricated using Anode with various sheet resistance. The discussion also concluded that the CNT based material attracts more bacteria and has shown excellent compatibility.	^5^
Bucky paper	4 µW/cm^2^	20 µA/cm^2^	Microfluidics-based MFCs is studied under the presence and absence of E. Coli. The work eliminated the need for a pre-treatment process and additive.	^6^
Carbon electrodes	1.34 µW/cm^2^	15.4 µA/cm^2^	Miniaturized Y-Shaped Microbial Fuel Cells are fabricated and studied for real-time long-time performance with the help of IoT-enabled devices.	^7^
Cellulose Fibre Paper	11.8 µW/cm2	91 µA/cm2	Filter Paper of different grade is used to understand and assess the performance of the MFCs. The electrodes conductivity is enhanced using metal based ink nanoparticles at the anodes.	^8^
Carbon Ink: MnO_2_	15.9 µA/cm^2^	130 µA/cm^2^	MFC is fabricated using the inject printing techniques, which is further modified with MnO2 to improve the electron transfer efficiency. The MFCs is used to power minituarized digital watch and can be further expanded for IoT applications.	^9^
Enzyme based screen printed electrodes	37.5 µW/cm^2^	–	Improvement in power density for both cathode and anode is observed with the introductions of enzymes, which overall improve the efficiency of the fuel cells.	^10^

As a quasi 0D carbon materials, Graphene Quantum Dots (GQDs) have a bonus over the traditional graphene-based material/2D Graphene and nanoparticles. GQDs have numerous active sites as compare to the 2D graphene which influences the edge defects, functional group, and doping^21^. GQDs also have an edge over nanoparticles as they possess and constitute better surface areas which favors microbial attachment, chemical stability, and faster electron mobility^22^. Boron doped GQDs also showed the enhancement of the conductivity of MFCs by resembling and forming the structure of nanoflowers in the active sites^23^. The active sites also contribute to improving the electrical conductivity^24^. Possessing such enhancing qualities GQDs have attracted a lot of interest in the scientific community.

In this work, the performance of electrodes using GSPEs technique is evaluated for unmodified MMFC and catalyst-based MMFC. Anode and cathode are fabricated using a microscopic glass substrate, polyvinyl chloride (PVC) Sheet, and carbon paste. Furthermore, the anode was enhanced with GQD implying that the valence electrons are highly delocalized over the entire domain area which results from the extensive sp^2^ hybridized carbons in crystalline graphene. GQDs provide a sp^2^ hybridization unique structure and larger active sites and allow more sites for biofilm formation and faster reaction kinetics boosting the overall performance of the MFCs as compared to the unmodified carbon pastes. Thus, GQDs are incorporated to improve the electrode characteristics. Electrochemical testing like Cyclic Voltammetry (CV), and polarization Studies are conducted for unmodified and GQDS-modified electrodes. The operational performance of both electrode types was evaluated also in MMFC.

The wastewater drained out of the Reverse Osmosis (RO) filter paper is used in the MMFC. The physicochemical testing properties of the wastewater such as Chemical Oxygen Demand (COD), Conductivity, and pH were measured to assess MFC performance. The results of this work demonstrate the performance of low-cost fabrication of electrodes for MMFC in the field of microfluidic devices, microelectronics, and Nano-electronics.

## Experimental detail

### Materials and instruments

Polyvinyl Chloride (PVC) sheet is purchased from Amazon.in (*e-commerce*). Carbon Pastes, Ag/AgCl Pastes, Isopropyl Alcohol (IPA), and Graphene-Quantum Dots are all purchased from Merck, and RO filter paper wastewater is collected from the laboratory purifiers waste filter paper. Fabrication of monolithic multilayer microfluidic devices (MMMD) from planar orientation (1 layer) to nonplanar (4 layers) monolithic microchannels has been previously presented which was found to be optimal for synthesizing highly potentialized silver nanoparticles (AgNPs) in less than a second^25^. The reason for using AgNps as a catalyst is to serve as a comparative baseline due to their conductive properties, despite their high antibiotic properties. The intent further infers that the MMFCs may derive its performance from planktonic cells as well rather than depending only on biofilm formations. This approach allows to distinguish and understand the role of catalyst by setting a baseline or reference study to it.

CO_2_ laser and engraver (VLS 3.60 with 30 W, 10.6 μm CO_2_ laser source, from Universal Lasers, AZ, US) is used to dissect the outline of the electrodes. A potentiostat (SP-150 from Biologic, France) with current resolution 760 pA is used for conducting different electrochemistry tests to analyze the performance of fuel cells, Liquid Conductivity meter (ELICO CM 180, India) is used to measure the conductivity of the wastewater used as the fuel, pH meter (pH Elico, India) is used to measure the pH of the wastewater, UV Spectrophotometer (Single Beam with Halogen Lamp, Spectroquant, Germany and Double Beam with Xenon Lamp, Spectroquant Prove 300, Germany), and Thermo Digestor (Spectroquent TR 320, Germany) are used to measure the Chemical Oxygen Demand (COD), Raman Spectroscope (Holmarc Confocal Laser Raman Spectrometer, India) is used to conduct the characterization properties of the electrodes and Scanning electron Microscope (Scientific Thermofisher model, USA) is used to study the surface morphology.

### Design and fabrication

**Fig. 1:**
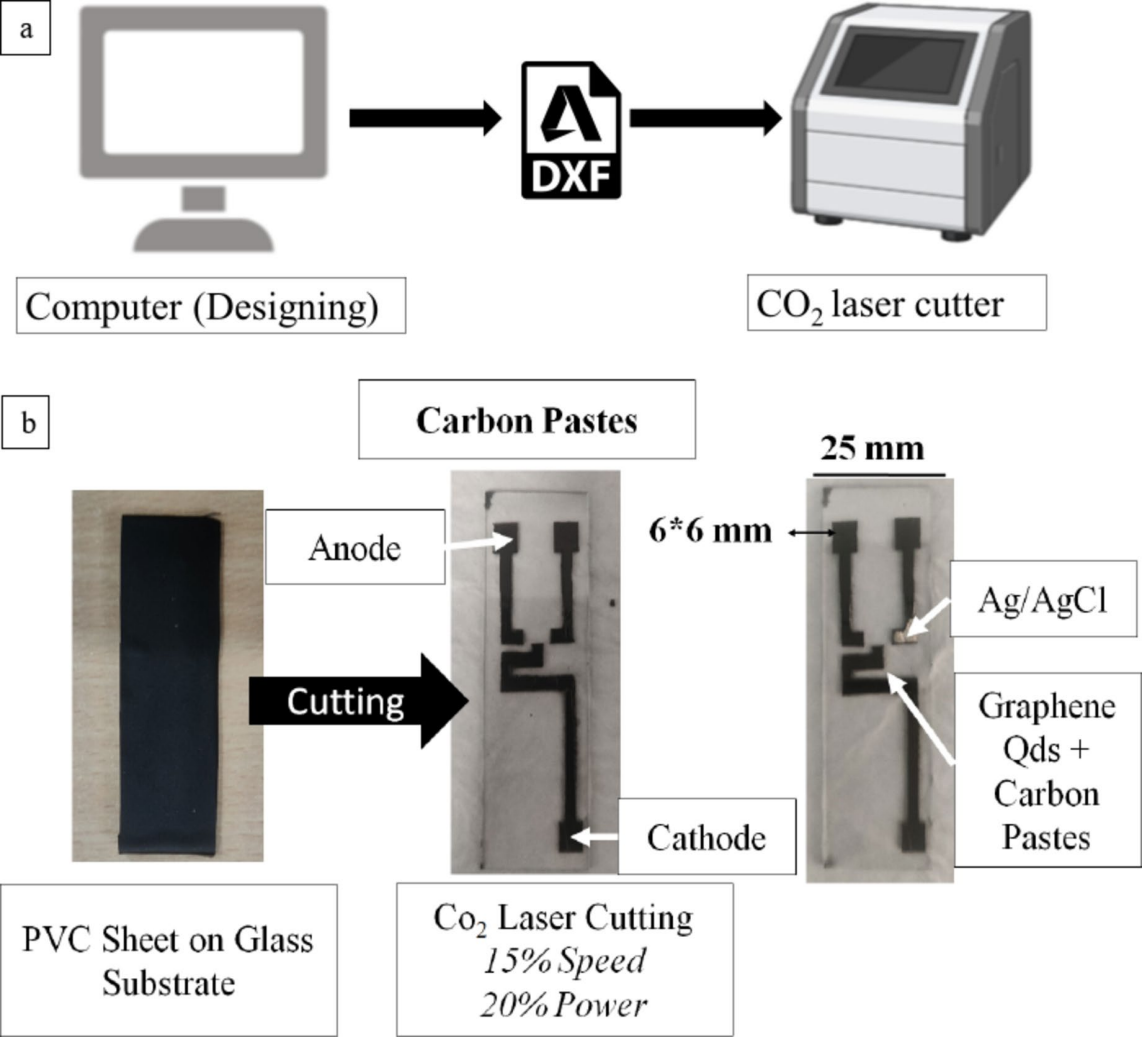
(**a**,**b**) Schematic of the GSPEs layout and fabricating/engraving with CO_2_ laser engraver.

AutoCAD/SolidWorks was used to make the manufacturing drawing of the electrodes. A microscopic glass layer of dimension of length 25 mm by breadth 75 mm is opted as the base of the operating electrodes. The identically sized PVC Sheet is layered on the top of the microscopic glass sublayer or base. The prepared sandwiched layer is further processed for engraving using a CO_2_ laser operated at 15% speed of 1200 mm/s and 20% power of 30 W. Figure 1 represents the schematic of the fabrication approach An AI picture generator (hotpot.ai/art-generator) is used to create the schematic image of the laptop.

Accomplishing and achieving the outline after the engraving of the fabricated microscopic layer, a layer of strokes of carbon paste painting is incorporated to achieve a uniform texture. An anode, a cathode, and a reference/assistance electrode are replicated by the intended shape of the designed electrodes. Ag/AgCl paste is painted over the reference/assisting electrodes for them to function as a supporting electrode for the cathode and anode during power/current overload. The fabricated Ag/AgCl layer is dried for an additional hour at 70 °C. The GSPEs painted in Carbon paste are henceforth fabricated and processed for further electrochemical testing.

The GSPEs painted in Carbon paste are henceforth fabricated and processed for further electrochemical testing. The corresponding Scheme also describes the concepts as mentioned above and displays the schematic approaches taken, along with the image of the manufactured GSPEs before their operation and testing.

Using a micro-pipette and drop casting approach over the anode, 5 µl GQDs are casted over the surface of the anode to obtain the second GSPEs which is further analyzed for additional comparative studies between all the prepared GSPEs layers.

Wastewater derived from the RO purifier was inoculated hereafter by drop casting using a micropipette in all three cells of MMFCs consisting of the unmodified, GQDs-based, and AgNps-based layers.

All the 3 layers of MMFCs are left to mature and stabilize under the controlled condition of 48–72 h, at room temperature of 24–30 °C and pH level of 7.5–7.8. This condition is crucial for optimizing and ensuring biofilm formation before further testing it.

The following inoculation is essential for addressing the electrochemical performance and overall working condition of the fuel cells in subsequent studies. The GSPEs layered MMFCs based on GQDs are further classified after the 5th operation, where a single operation is defined as the complete cycle of MMFCs performance assessment which includes inoculations and electrochemical testing. Between operations, a simple inoculation is performed just by soaking out and drop-casting the inoculation in GSPEs layered MMFCs without an additional cleaning process. This approach has been consistent for all repetitions including the MMFCs post-5th operations.

## Results and discussion

### Electrode characterizations

**Fig. 2:**
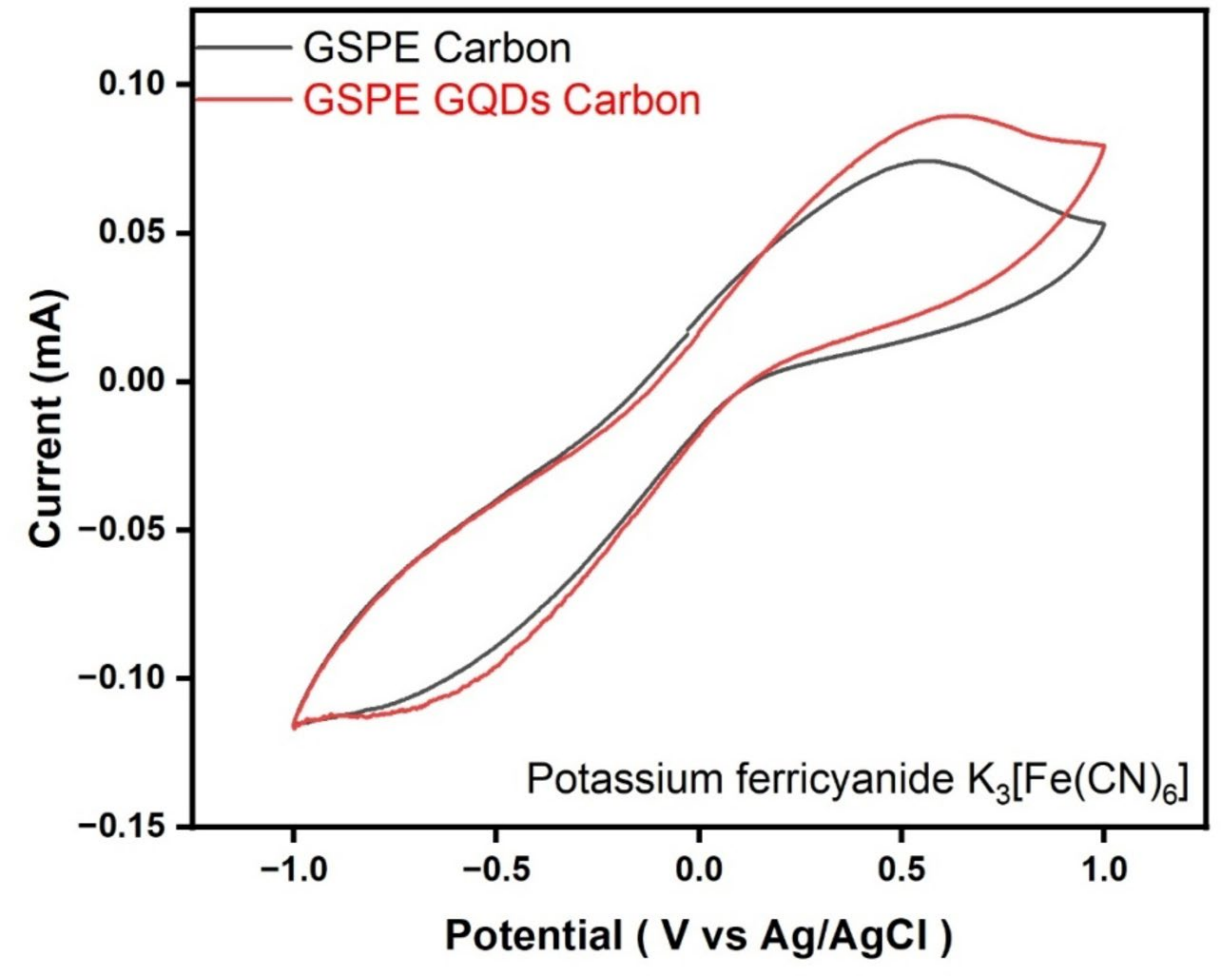
Cyclic Voltammetry of K_3_[Fe(CN)_6_] scanned in the potential window of 1.0 V to – 1.0 V with the scan rate of 50 mV/s for 10 cycles.

Electrodes are tested using Potassium Ferricyanide [K_3_Fe(CN)_6_] Cyclic Voltammetry (CV) is performed and carried out to obtain the standard curve of the carbon paste-based electrodes. The potential window is scanned for 10 Cycles at 50 mV/s while being fixed at 1.0 V to – 1.0 V. The choice of the last scan ensures that any initial transient or conditioning effects are minimized providing a more accurate analysis of the system performance in a stabilized state. An active reaction between electrode and electrolyte interface is displayed by the active oxidation peak as shown in Fig. 2. While a very minimal reduction peak is noticed. The obtained peak for GSPEs indicates the working condition and operational state of the electrode. The peak of the CV curve also often indicates the amount of concentration, electrode kinetics, and redox activity. Thus, before testing the electrodes painted in the GSPEs with the working electrolyte it is crucial and essential to test them in the standard solution to understand the dynamics of the electrolytes that is utilized as the fuel of the GSPEs.

### Physico-chemical characterizations

#### Chemical oxygen demand (COD)

The RO purifier water in the volume of 3 ml, derived from the RO purifier wastes, is analyzed for Chemical Oxygen Demand (COD) with two different COD solutions consisting of 0.30 ml of COD (A) and 2.85 ml of COD (B) prepared for the measurement parameters of 4.00–40.00 mg/l of concentrations. The solution is digested in the thermal reactor digestor for 2 h at 150 °C. The solution is further tested using two different UV-spectrophotometers to verify the recorded output. The spectrophotometer Single-Beam based halogen lamp displayed a similar output of less than or equal to 4.00 mg/l in comparison to the Double-Beam based xenon lamp which displayed an output of less than or equal to 4.00 mg/l too. The absorbance of the recorded solution is measured around 1.65. The recorded data in minimal range can be attributed to weaker concentration of organic compounds which will significantly contribute to lesser fouling/corrosion and decreasing the maintenance required hereby improving the repeatability of the GSPE for MMFCs operations.

#### pH analysis

The pH measurement is carried out to determine the favorable range for microbial community and to prevent constraints of electrode fouling/corrosion. MMFCs have shown a specific range of pH where they produce sufficient current and give stable performance consistently. Such pH is recorded around 7.66 which comes under the optimal range of biofilm formation of 6–9 as per the survey conducted by Rickelmi et al.^26^. The activities in the range of 7–8 pH can be attributed to the increased utilization of proton rather than their liberation during the electrochemical kinetics among microbial activity at the anode making a suitable domain for operations^27^. The pH was measured before using it in the electrochemical testing for GSPEs-based MMFCs and is not adjusted throughout the experiments. This value displays the pH of the RO wastewater used as the inoculum. Finally, the conductivity of the sample is measured around 0.55 Ω^−1^ cm^− 1^ to determine its contribution, as most ions and microorganism were eliminated during the process of reverse osmosis occurring in the chamber of waste filter paper. The electrolytes conductivity showed an inadequate value. The obtained information illustrates an overview of the significant impact of carbon paste and GQDs.

#### Raman spectroscopy

**Fig. 3:**
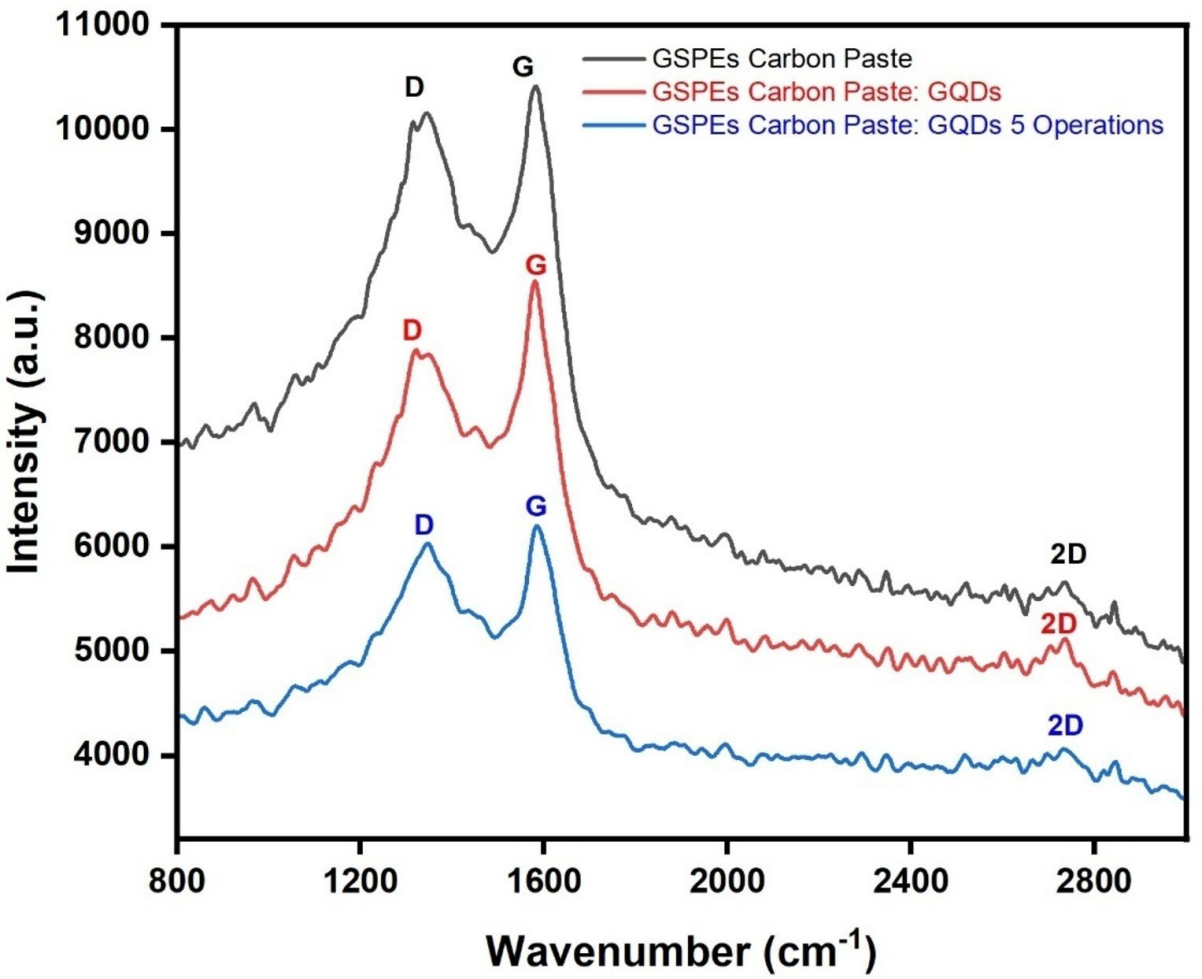
Raman Spectrum of Carbon based GSPEs, GQDs coated GSPEs and post 5 operation GQDs based GSPEs.

Raman Spectroscopy is conducted to execute a thorough examination of the prepared GSPEs based MMFCs and GQDS coated GSPEs to confirm the formation and absence of Graphene based materials. The result is obtained after optimizing the laser power to 1.2–1.8 W and speed around 0.580 cm/s and plotted as shown in Fig. 3. As observed from the graph, three prominent Raman peaks of D, G and 2D is obtained. Close to similar optimization for graphene-based substrate is conducted by Rewatkar et al.^28^.

The three different layers of GSPEs i.e. Carbon Paste based, GQDs Carbon Paste based, and GQDs Carbon paste-based post 5th operation is measured individually, to obtain the I_D_/I_G_ and I_2D_/I_G_ ratios as shown in Table 2.

**Table 2:** The I_D_/I_G_ and I_2D_/I_G_ ratios obtained from the Raman Spectra.

No	Sample Name	I_D_/I_G_	I_2D_/I_G_
1	Carbon Based GSPEs	0.97	0.65
2	GQDs coated Carbon Based GSPEs	0.92	0.60
3	Post 5th Operation GQDs coated Carbon Based GSPEs	0.98	0.54

The degree of defects is obtained by I_D_/I_G_ while the I_2D_/I_G_ explains the graphitic structure sp2 c = c and hole mobility. Less defects is indicated by minimal number of I_D_/I_G_ while higher charge mobility is indicated by higher value of I_2D_/I_G_^28^. As observed from the table GQDs coated GSPEs showed the least defect while the post 5 operated GSPEs showed the highest defect. By comparing the charge mobility too, it can be noticed that unmodified Carbon paste showed significantly better mobility as compared to the GQDs modified layers. Despite having high defects, the unmodified GSPEs showed better mobility, this may be due to the non-uniform distribution of the defects which may have created an unusual pathway that facilitated easier movement for the charge carriers. This explanation could account for the unusual observation of higher mobility despite displaying higher defects.

#### FT-IR spectrum

**Fig. 4:**
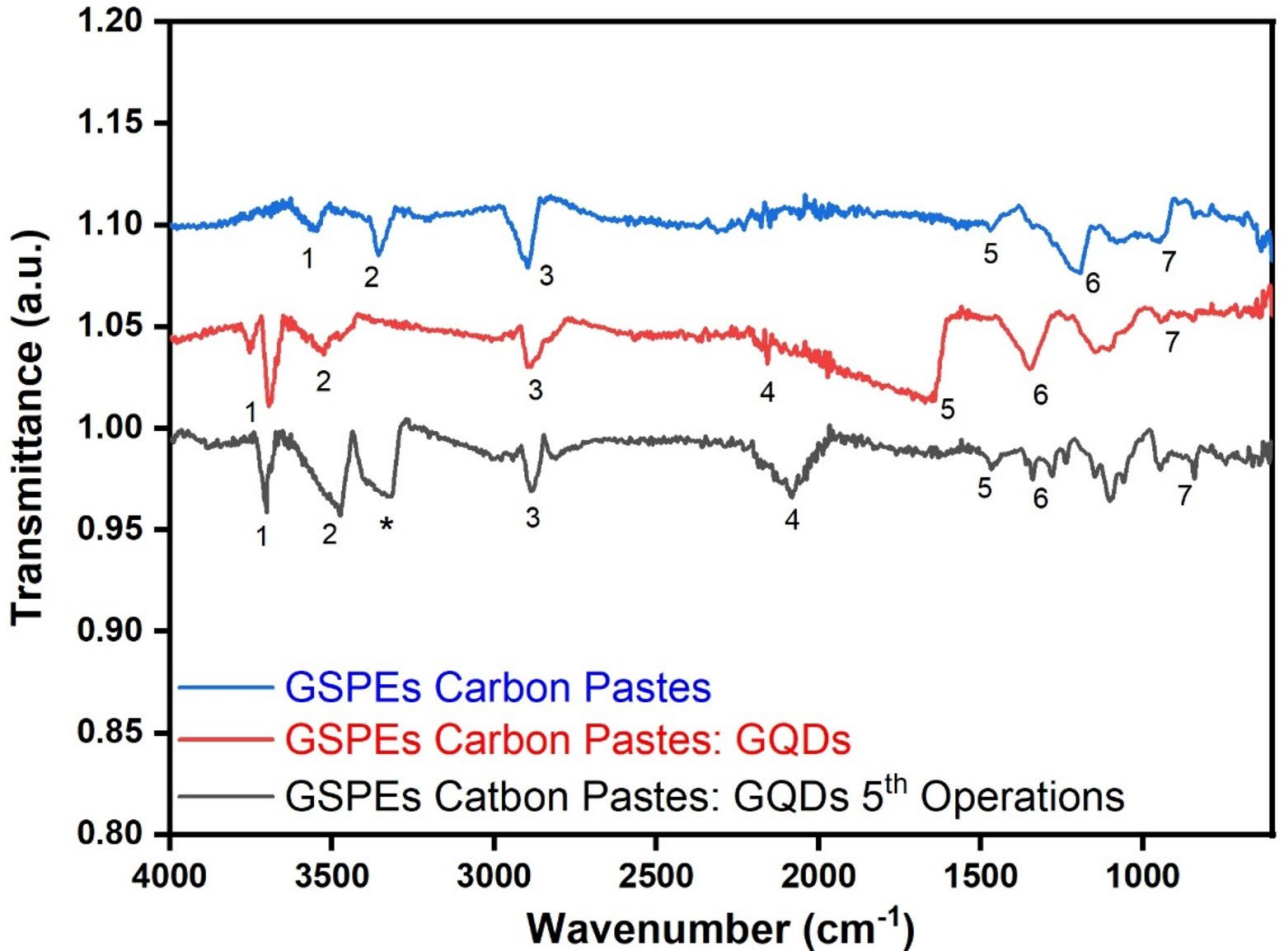
FT-IR spectrum of GSPEs based layers, QDs painted GSPEs based layers, and QDs painted GSPEs post 5th operations.

Fourier Transform Infrared (FTIR) Spectroscopy revealed the functional group composition of the GSPEs (Fig. 4). Various absorption bands are obtained for the significant GSPEs based layers i.e. Carbon paste based GSPEs, GQDs coated carbon paste based GSPEs, and post 5th operated GSPEs.

As observed in the FT-IR graph in Fig. 4. the absorption spectra recorded in the unaltered GSPEs showed the absorption spectra at 3524.49 cm^− 1^, 1672.11 cm-^1^, and 1147.77 cm-^1^ O-H stretch, C = O stretch, and C-O stretch. When the GSPEs are altered with QDs the shift significantly shifted to 3475.08 cm^− 1^, and 1102.78 cm-^1^ for the corresponding groups, while the peak at 1672 cm^− 1^ disappeared. An additional peak is observed at 3316.08 cm^− 1^ and 2083.06 cm^− 1^ designating the formation of C-H stretch in the Alkynes group and R-N = C = S structure indicating the modification of the carbon paste-based GSPEs layers ^29 30 31^. The additional functional group acts as an electron donor/mediator facilitating increased charge carrier ability and mobility, initiating larger electron transfer mechanism which influences the power output and efficiency of the MMFCs.

#### Morphological studies with SEM (scanning electron microscopy)

**Fig. 5:**
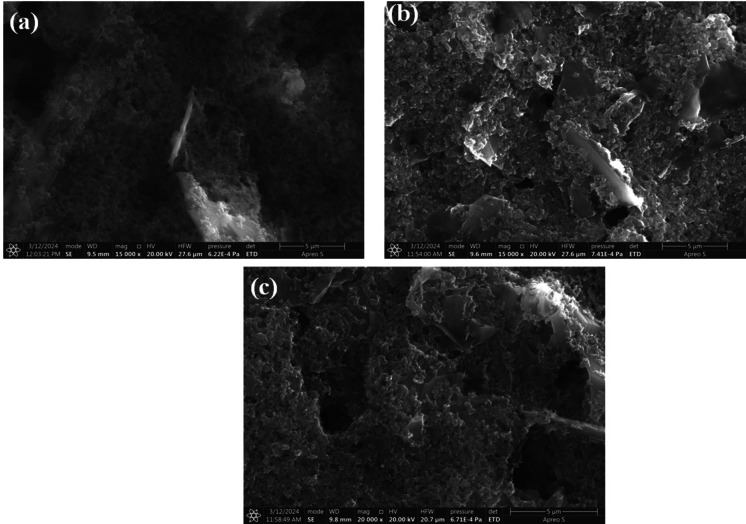
SEM micrograph images of GSPEs layers (**a**) unaltered substrate based on carbon pastes (**b**) GQDs painted carbon layers (**c**) Post 5th operation GQDs based GSPEs layers.

The morphological structure of carbon-based GSPEs and GQDs modified GSPEs layers is examined using high resolution Scanning Electron Microscopy (SEM). As observed from the Fig. 5. above SEM micrograph images of GSPEs based carbon paste showed a structure of fracture surfaces while the GQDs painted Carbon pastes based GSPEs displayed a mixture of semi-fracture and semi-porous structure, which felicitates room for biofilm formation necessary for the kinetics between the electrodes and the microorganisms. The change in morphological structure observed between the unaltered Carbon Paste based GSPEs and GQDs painted Carbon paste based GSPEs indicated the presence of GQDs. Figure 5c displays the morphological characteristics of the GQDs modified GSPEs to analyze the presence of GQDs after 5 operations. As observed from the morphological structure, there is a decline in the visibility of semi-porous semi fractured state. However, because of the exhibition of semi-porous structure which were absent in the unaltered carbon based GSPEs layers, it can be concluded that there is a good amount of GQDs retained even after repeating the test 5 times which clarifies the reasoning behind enhanced repeatability. Consequently, the measuring parameters for the corresponding layers is measured around 5 μm at magnification of 15,000 X and HV of 20.00 kV. The insights of the morphological analysis help in optimizing the GSPEs further.

### Electrochemical studies

#### Cyclic voltammetry

**Fig. 6:**
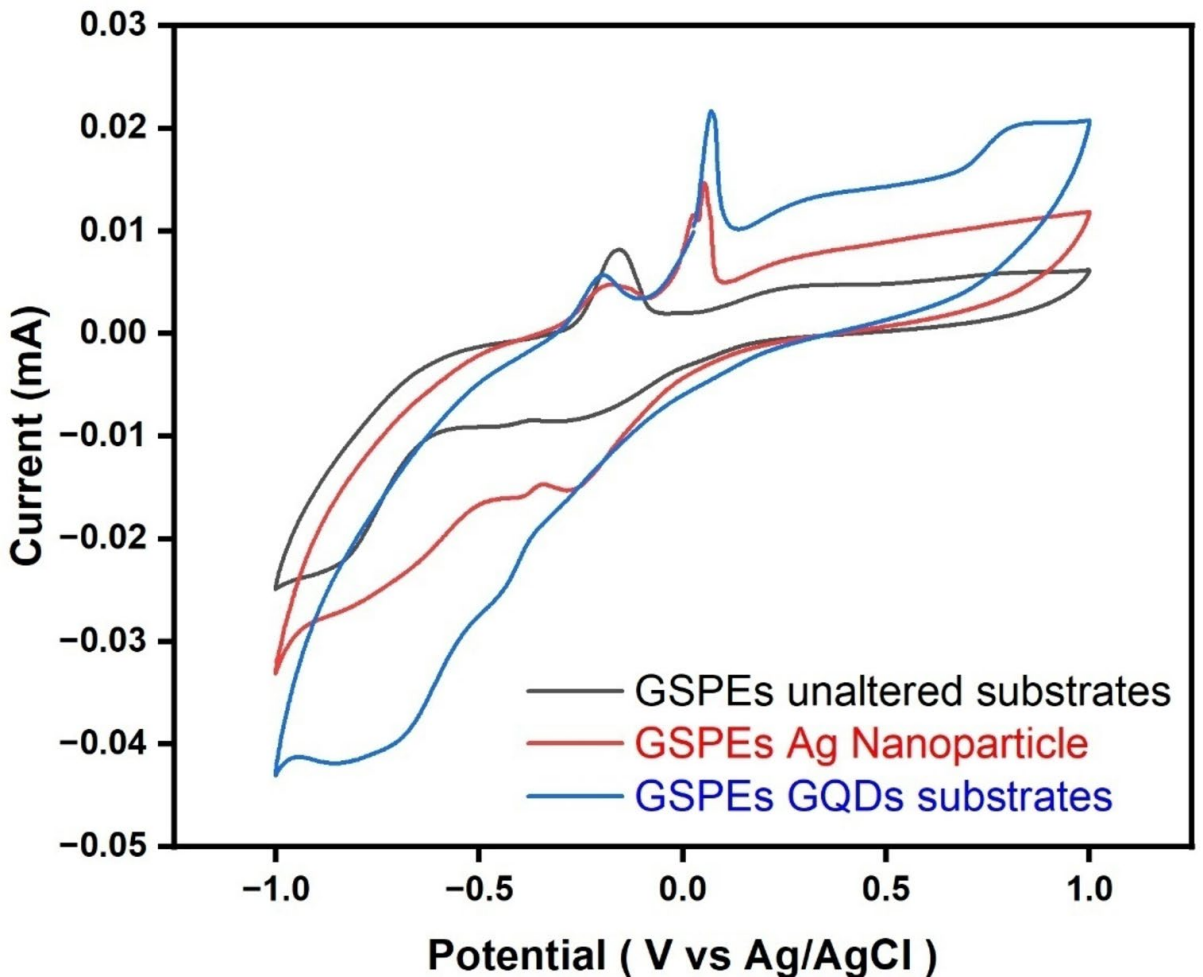
Cyclic Voltammetry of RO filter-paper wastewater scanned at the potential window of 1.0 V to – 1.0 V with the scan rate of 20 mV/s for 10 cycles.

When comparing the surface of carbon-pasted GSPEs covered in GQDs with the unaltered surface of carbon paste. Unmodified Carbon paste displayed a weaker peak, showing a weaker reaction between the electrodes and electrolyte interfaces. The role of GQDs plays a considerable part in the reaction kinetics between the electrodes and electrolytes due to the unique property of GQDs. GQDs provide smaller dimensions and higher surface area as compared to the commercial carbon pastes resulting into more active sites essential for the active electrochemical interaction of molecules. To further confirm the response of GQDs the layers is compared to the identical layers fabricated of AgNps. As observed, AgNps provided a response with a stronger peak in comparison to the unmodified GSPEs-based layer while a weaker response in comparison to the GQDs-based GSPEs layers. Further, confirming the influence and response of GQDs. The potential window for both layers is fixed between 1.0 V to -1.0 V and is operated at scan rate of 20 mV/s for 10 cycles. As seen from Fig. 6. when GQDs are deposited on the layer a tiny peak can be detected as compared to the small unnoticeable peak in the unmodified surface of the carbon pastes. From the recorded data, it can be herby concluded that the introduction of GQDs in the surface of carbon pastes is enhancing the electrochemical reaction kinetics between the electrodes and electrolytes interface improving the overall efficiency of the fabricated MMFCs. The spike in oxidation peaks can be attributed due to the improved surface area contributed by the catalysts used to provide a greater number of active sites for oxidations during electrochemical kinetics, leading to prominent oxidation peaks. Additionally, the presence of the residual organic compound in the RO wastewater may also contribute to the observable peak.

#### Polarization studies

**Fig. 7:**
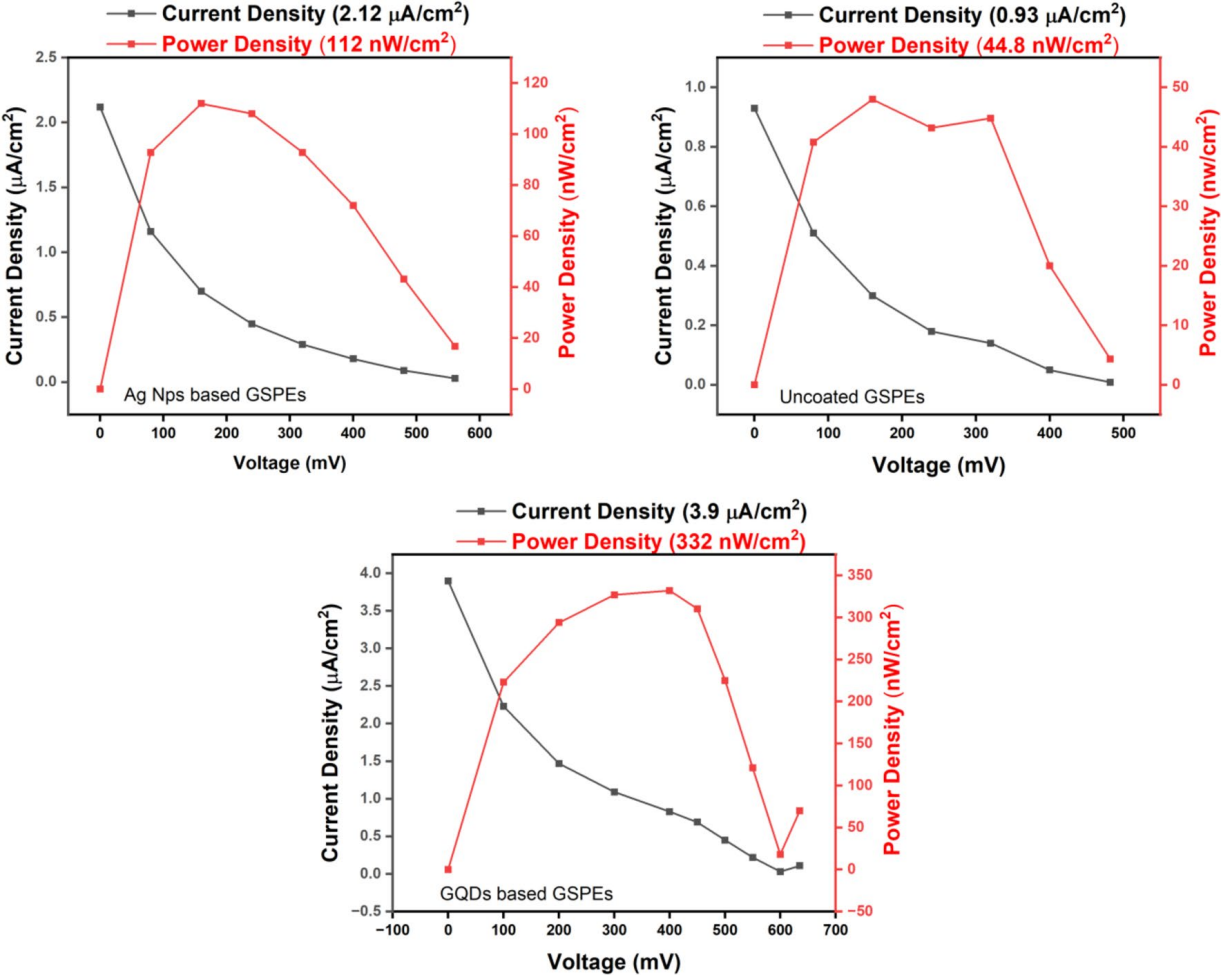
Polarization Studies of (**a**) Unmodified Carbon Paste GSPEs based microscopic layers (**b**) GQDs painted Carbon Pasted based GSPEs layers.

As stated earlier the overall goal of the study is to evaluate the effect of GSPE on the power output of a MMFC. As seen from Fig. 7, the unaltered carbon pastes displayed a current density of 0.93 µA/cm^2^ and a power output of 44.8 nW/cm^2^. Despite showing a low conductivity and minimal COD, the GSPEs generated a high current because of the influence of the catalyst used which improve the relationship between the microorganism activities which enhanced the electron transfer mechanisms between the electrodes to provide better pathway necessary for conduction of electrons towards the outward circuit. While the unmodified GSPEs showed relatively poorer performance due to the absence of the catalytic properties.

The GQDs modified layer presented a current density of 3.9 µA/cm^2^ and a power density of 332 nW/cm^2^ because of GQDs characteristics of sp^2^ hybridizations which formulate a chemistry of π-electron system and hexagonal lattice structure that maximizes the potential of electrochemical kinetics and contributes to a very high electrical conductivity ^32–34^. This characteristic also helps in the stimulation of bio-film development, which improves the bacteria attachment in the electrode surfaces that will further help in the conduction of the electrons necessary to generate the required energy. According to the above comparative analysis, it is observed that the output obtained for GQDs painted layers outperformed the data of unaltered carbon pastes. The recorded result is further verified by comparing the GQDs based layer with a dummy layer coated in AgNps which showed the response of 2.12 µA/cm^2^ and 112.39 nW/cm^2^ of current and power density respectively further verifying the outcomes of GQDs and its subsequent characteristics and electrochemical properties. The obtained data is summarized in Table 3.

**Table 3:** Comparative tabulated performance data of the unaltered Carbon Paste based GSPEs, Carbon Paste-AgNps-based GSPEs, and GSPEs carbon-based GQDs.

No	Layers Name	Open Circuit Potential (mV)	CD (µA/cm^2^)	PD (nW/cm^2^)
**1**	Carbon GSPEs	482	0.93	44.80
**2**	Ag Nps-GSPEs	561	2.12	112
**3**	GQDs GSPEs	635	3.90	332

#### Electrochemical impedance spectroscopy (EIS)

**Fig. 8:**
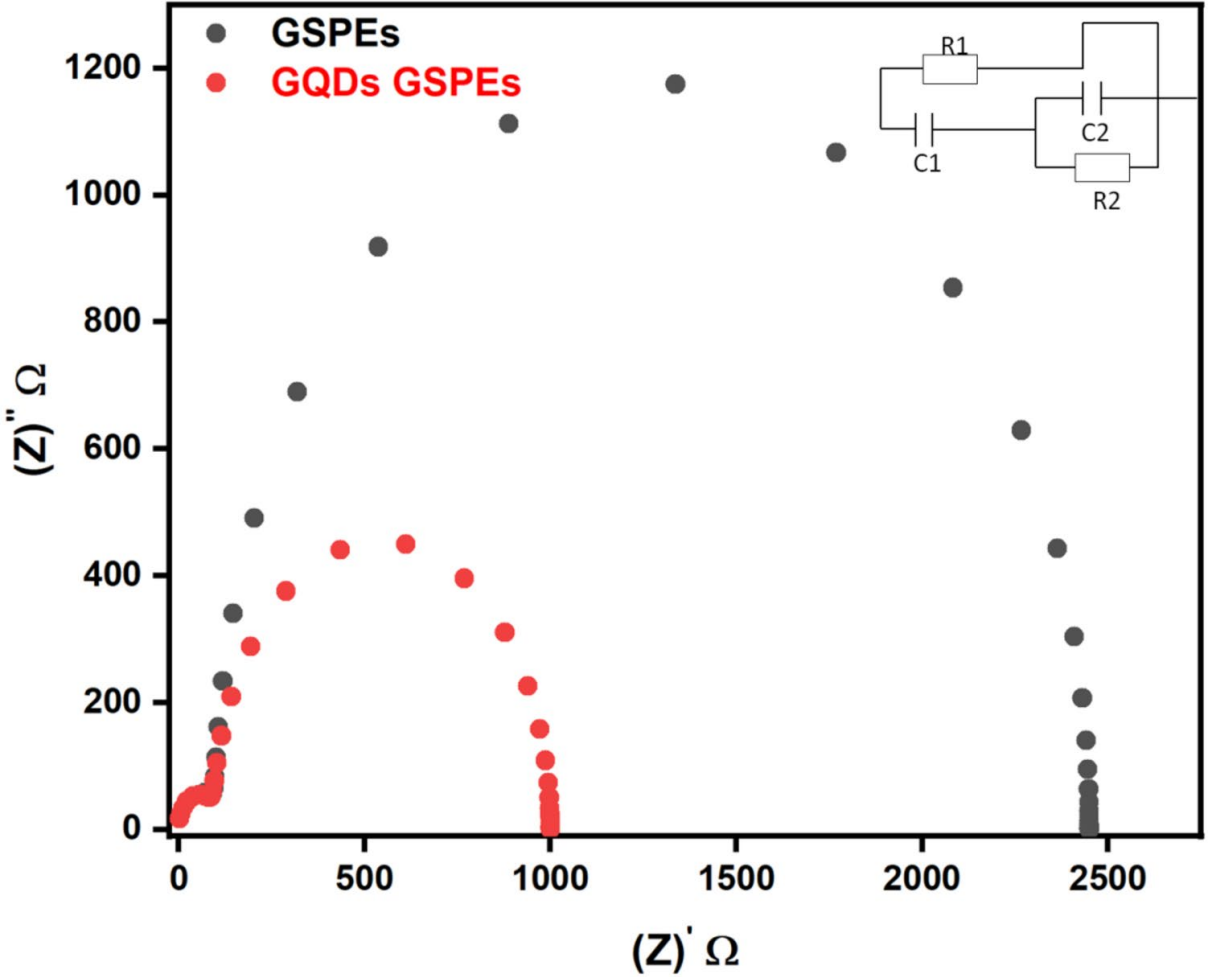
Nyquist Plot data of unaltered Carbon pastes layers and GQDs painted Carbon pastes-based layers.

Electrochemical Impedance Spectroscopy (EIS), data is presented referred to as the Nyquist plot, and is useful to provide insights to the charge transfer resistance R_CT_, which is the resistance between electrode surface and electrolytes. It is often associated with the kinetics of the electrochemical reaction occurring at the electrode surface. As observed from Fig. 8. the unmodified layers of GSPEs displayed a higher charge resistance value as compared to the GSPEs modified substrates with GQDs. The circuitry of the is displayed in Fig. 8. and the Nyquist graph is obtained by plotting the Randle Equivalent Circuit corresponding to R1/(C1 + C2/R2). As observed from the recorded data and the obtained graph the GQDs modified GSPEs layers showed a relatively lower R_CT_ of around 1 KΩ in comparison to the unaltered GSPEs layers which recorded a high resistance of 2.48 KΩ. GQDs have shown lesser resistivity further proving the quality of the modifications this may be due to the additional functional group observed via FT-IR graphs which allows extra electron carriers, high mobility of charge carrier obtained via I_2D_/I_G_ observed in Raman Shift, and the morphological structure allowing better Enhanced Electron Transfer (EET). In Conclusion, the layers based on GQDs GSPEs show better performance due to higher charge carriers and comparatively lower resistivity.

### Electrochemical studies

The GSPEs capacity to consistently demonstrate a similar performance throughout multiple runs for the MMFCs application can be determined by the repeatability tests. (See Table 4.)

The percentage value mentioned, refers to the peak power value obtained from the polarization curves. These values are taken into consideration to assess the electrochemical performance across different parameters and conditions.

**Table 4:** Comparative tabulated data of the unaltered Carbon Paste-based GSPEs and GSPEs Carbon Paste based GQDs.

No	No. of Operations	GSPEs Carbon Pastes %	GSPEs GQDs-Carbon pastes%
1	First	100	100
2	Second	79.15	99.41
3	Third	60.37	99.39
4	Fourth	52.35	96.01
5	Fifth	47.51	95.98

**Fig. 9:**
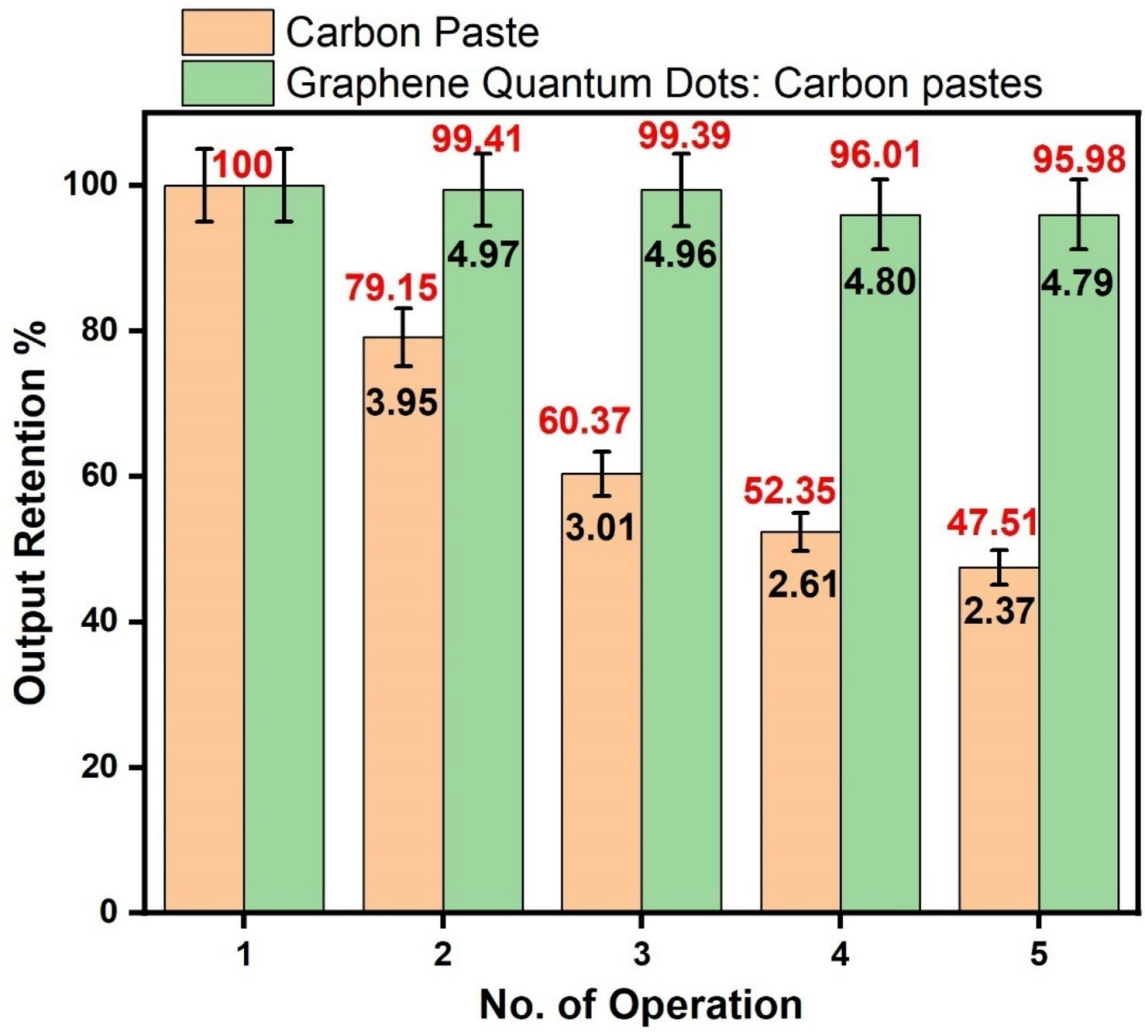
Performance repeatability of MMFCs with unmodified anode and anode modified with GQDs.

Figure 9 Illustrates how the GQDs sandwiched GSPEs layers demonstrated a more stable output for the 5-operation conducted as compared to the atrophy observed in the unaltered layers of the GSPEs painted in carbon pastes. This may be due to the composition of wastewater derived from the RO Purifier used as the fuel. The combinations of microorganisms and the possible presence of ions can alter the chemical composition of carbon pastes more as compared to GQDs doped Carbon pastes influencing and affecting its surface properties resulting in biofouling and adversely affecting its conductivity and long-term performance deteriorating the general repeatability of the unmodified carbon-based layer, while GQDs provide a protective barrier over the carbon paste hence delaying the fouling process.

As observed in Fig. 9; Table 4, GQDs displayed better repeatability after the 5th operation retaining 95.98% of the output obtained in the 1st operation. In contrast, the unaltered Carbon-based GSPEs obtained only 47.51% of the total output in the 5th operation as compared to the output obtained in its first operation. This may be due to the unique property of GQDs which gives an additional active site that improves the overall surface area which is essential for the electrochemical reaction between the microbes and other contributing parameters ^35–38^. The purpose of adding GQDs to the layered Carbon GSPEs is the ability to make them more durable for single use and improving the power output compared to the unmodified version of GSPEs. Additionally, GQDs displayed enhanced performance due to their nanostructured nature, offering large surface area to improve charge transfer at the anode. GQDs also provided better chemical stability and resistance to degradation to increase life span of anodes. The data and the GSPEs-based GQDs after the 5th operation are used as the “post 5^5h^ operations” in the characterization sections to justify and understand the overall influences of GSPEs layered GQDs and unmodified GSPEs in the overall performance of MMFCs. This ensures clarity in the data presented and its interpretations.

## Conclusions

In this paper GSP technique for manufacturing electrodes is presented and tested in MMFCs for its performance. The wastewater used in the MMFC was taken from the waste filter of RO purifier with pH around 7.66, the conductivity of around 0.55 Ω^-1^ cm^-1^ and the COD was approximately 4.00 mg/l. The tests were run with anode modified with GQD and performance was compared with reference anode modified with Ag-Nps. The experimental studies led to the following conclusions.

- Overall, the glass screen-printing (GSP) technique is a reliable approach as glass-based layers are less susceptible to biofouling/corrosion when in contact with microorganisms as compared to the traditional electrodes. However, the GSPEs was found to have low repeatability, as the performance of the cell deteriorated up to around 47.51% after 5th operations. It was observed that some fouling occurred after repeated operations. Whereas the use of GQDs on the anode modified the stability of the surface layers and improved repeatability by up to 95.98% after 5th operation.
- The GQD modified anode also results in a better overall performance as observed in increased power output compared to that for non-modified anode. The graphene quantum dots offer more active sites at the surface and improve the conductivity of anode for better electron transfer. GQDs-based GSPEs showed seven times higher power output 332 nW/cm^2^ compared to its unaltered electrode which displayed a power output of 44.8 nW/cm^2^.
- The power output of GQD modified anode at 332 nW/cm^2^ was than 33.7% better compared to the anode doped with Ag-NPs which displayed 112 nW/cm^2^. Therefore, the GSP modified with GQD presents an alternative approach for making electrodes for MMFCs which is cost effective and shows overall good performance.

## Data Availability

The datasets used and analysed during the current study are available from the corresponding authors upon reasonable request.
